# Skin Lesions in the Upper Lip/Nasal Region

**Published:** 2011-01-12

**Authors:** Alexis C. Lanteri

**Affiliations:** Department of Surgery, Division of Plastic Surgery, UMDNJ—New Jersey Medical School, Newark, NJ

## DESCRIPTION

Case 1: A 50-year-old woman presents for excision of lesion along right nasolabial fold, which has been present for 2 years and has been increasing in size. Patient describes the lesion as itchy and denies pain, bleeding, or other symptoms.

Case 2: A 47-year-old man presents for excision of lesion along right nasolabial fold, which has been present for 1.5 years and has been increasing in size. Patient describes the lesion as itchy and occasional bleeding. He denies pain or other symptoms.

## QUESTIONS

1. **Describe the lesion in each picture.**
2. **What are the differential diagnoses?**
3. **State the various treatment modalities of these lesions.**
4. **What reconstructive options are available?**

## DISCUSSION

There is a broad differential diagnoses for lesions in the nasal region, which includes traumatic injury to the skin (sunburns), benign conditions (fibrous papule, seborrheic keratosis), precancerous (actinic keratosis) and cancerous (squamous cell carcinoma, keratoacanthoma, or basal cell carcinoma [BCC]) lesions. A full patient history is important to narrow the diagnosis and biopsy is critical to identify potential neoplasia.

In the 2 cases described, biopsy identified both lesions as BCC. Basal cell carcinoma is the most prevalent skin cancer and is commonly found in sun-exposed areas including the head and neck. Ultraviolet light causes formation of thymine dimers and depression of the local immune system, allowing pluripotent cells in the basal layer of the epidermis to proliferate uncontrollably.

Once diagnosed, the goal of treatment is eradication of the tumor to prohibit further malignant proliferation. Surgical treatment options for BCC include curettage and electrodesiccation, Mohs procedure, and excision. Factors such as tumor size and location, patient age and ability to tolerate surgery, and expense must be considered. Curettage and electrodesiccation is quick and inexpensive with high cure rates. However, it is a blind technique and this lack of margin control limits its use to nonaggressive and superficial BCC. Mohs surgery examines 100% of surgical margins to provide the best long-term cure rates; however, it is expensive and time-consuming when compared to other techniques. Surgical excision removes the clinically apparent tumor and a sufficient margin to achieve high cure rates. For patients who have multiple comorbidities or cannot tolerate anesthesia, cryotherapy and radiation therapy are alternative treatment options.

In determining the best surgical margin for BCC, Gulleth et al conducted a meta-analysis of the literature and compared average recurrence rates and the relative risk of recurrence in margins 1- to 4-mm versus a 5-mm resection. Although a larger surgical margin lowers the relative risk of recurrence, they found that achieving a clear surgical margin does not correlate with lower recurrence risk. For surgeons who desire a minimum 95 percent cure rate, the data indicates that a 3-mm surgical margin may be safely used for BCC lesions 2 cm or smaller.

The 2 modalities considered for reconstructive management in these patients are full-thickness skin grafts (FTSG) and transposition flaps. FTSG are a single-stage procedure, they preserve the anatomy and tend to have smaller incision lines. Transposition flaps like the V-Y advancement flap are alternatives to FTSG and take little time, are not risky, and recover faster. However, planning must be meticulous or a bad outcome will result. Variables such as the size of the defect, its location, the quality of skin, and the surgical preference are used to determine the best treatment for each individual patient.

## Figures and Tables

**Figure:**
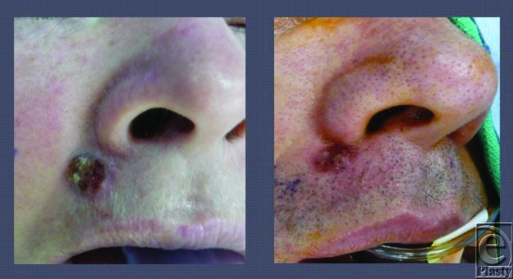


**Figure 1:**
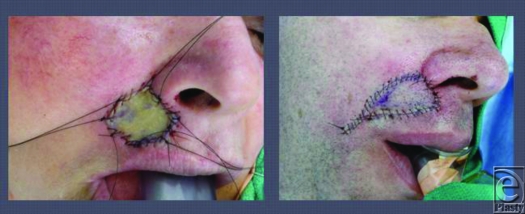
Full-thickness skin grafting (*a*) and V-Y advancement flap (*b*).
